# Comparison of a 3D CZT and conventional SPECT/CT system for quantitative Lu-177 SPECT imaging

**DOI:** 10.1186/s40658-024-00627-1

**Published:** 2024-03-19

**Authors:** Victor Nuttens, Georg Schramm, Yves D’Asseler, Michel Koole

**Affiliations:** 1 Nuclear Medicine, OLV Aalst, Aalst, Belgium; 2 Nuclear Medicine and Molecular Imaging, KU Leuven, Leuven, Belgium; 3 Nuclear Medicine, Ghent University Hospital, Ghent, Belgium; 4 Diagnostic Sciences, Ghent University, Ghent, Belgium

**Keywords:** Nuclear medicine, Dosimetry, Quantitative SPECT

## Abstract

**Purpose:**

Next-generation SPECT/CT systems with CdZnTe (CZT) digital detectors in a ring-like setup are emerging to perform quantitative Lu-177 SPECT imaging in clinical routine. It is essential to assess how the shorter acquisition time might affect the image quality and uncertainty on the mean absorbed dose of the tumors and organs at risk compared to a conventional system.

**Methods:**

A NEMA Image Quality phantom was scanned with a 3D CZT SPECT/CT system (Veriton, by Spectrum Dynamics) using 6 min per bed position and with a conventional SPECT/CT system (Symbia T16, by Siemens) using 16 min per bed position. The sphere-to-background ratio was 12:1 and the background activity concentration ranged from 0.52 to 0.06 MBq/mL. A clinical reconstruction protocol for dosimetry purposes was determined for both systems by maximizing the sphere-to-background ratio while keeping the coefficient of variation of the background as low as possible. The corresponding image resolution was determined by the matching filter method and used for a dose uncertainty assessment of both systems following an established uncertainty model..

**Results:**

The optimized iterative reconstruction protocol included scatter and attenuation correction for both systems and detector response modeling for the Siemens system. For the 3D CZT system, 6 iterations and 8 subsets were combined with a Gaussian post-filter of 3 mm Full Width Half Maximum (FWHM) for post-smoothing. For the conventional system, 16 iterations and 16 subsets were applied with a Gaussian post-smoothing filter of 1 mm FWHM. For these protocols, the sphere-to-background ratio was 18.5% closer to the true ratio for the conventional system compared to the 3D CZT system when considering the four largest spheres. Meanwhile, the background coefficient of variation was very similar for both systems. These protocols resulted in SPECT image resolution of 14.8 mm and 13.6 mm for the 3D CZT and conventional system respectively. Based on these resolution estimates, a 50% dose uncertainty corresponded to a lesion volume of 28 mL for the conventional system and a lesion volume of 33 mL for the 3D CZT system.

**Conclusions:**

An optimized reconstruction protocol for a Veriton system with 6 min of acquisition time per bed position resulted in slightly higher dose uncertainties than a conventional Symbia system using 16 min of acquisition time per bed position. Therefore, a 3D CZT SPECT/CT allows to significantly reduce the acquisition times with only a very limited impact on dose uncertainties such that quantitative Lu-177 SPECT/CT imaging becomes much more accessible for treatment concurrent dosimetry. Nevertheless, the uncertainty of SPECT-based dose estimates remains high.

## Introduction

Prostate cancer (PCa) is the second most frequent malignancy worldwide, after lung cancer [1]. Promising results have been reported in patients with Metastatic castrationresistant prostate cancer (mCRPC) treated with Prostate-specific membrane antigen (PSMA) Radio-ligand therapy (RLT) [2, 3]. More specifically, the $\beta$-emitting Lu-177-PSMA-617 has been evaluated extensively in the TheraP [4] and VISION trial [5], two randomized controlled trials demonstrating that treatments combining Lu-177-PSMA-617 with standard care significantly prolonged progression-free survival and overall survival for mCRPC patients compared to standard treatment.

RLT with Lu-177-PSMA-617 generally consists of a fixed treatment scheme with up to 6 cycles of 7.4 GBq Lu-177-PSMA-617 administered intravenously once every 6 weeks. During treatment, Quantitative Single Photon Emission Computed Tomography (QSPECT) can be performed to estimate the absorbed doses to tumors and Organs-at-risk (OAR). Recently, next-generation SPECT/CT systems with swiveling CdZnTe (CZT) digital detectors in a ring-like setup are emerging to perform Lu-177 QSPECT in a clinical setting. While a conventional SPECT/CT system uses two flat detectors consisting of a NaI(Tl)-crystal and photomultipliers to localize incident photons based on the visible light generated by the crystal and localized by Anger electronics, a 3D CZT SPECT/CT system uses semiconductors to directly convert incident photons into an electric signal. This direct conversion is supposed to result in superior sensitivity, spatial and energy resolution compared to a conventional system [6]. However, the energy range of the Veriton, the 3D CZT system from Spectrum Dynamics, is limited for SPECT measurements because of the thickness of the crystal. The system is designed for optimal detection of low-energy gamma photons (up to 200 keV) and comes equipped with a corresponding, non-exchangeable collimator. The collimators of a conventional system can be exchanged depending on the energy of the gamma photons to be detected. As a result, the conventional system can use the 208 keV photopeak for Lu-177 QSPECT imaging while the Veriton CZT system uses an energy window centered around the 113 keV photopeak.

Because of these differences in system technology and acquisition protocols, a comprehensive comparison between a 3D CZT and conventional Anger-type SPECT/CT system is essential to evaluate Lu-177 QSPECT imaging performance and assess how the shorter acquisition time with a 3D CZT SPECT/CT system affects the accuracy and precision of the mean absorbed dose estimates for tumors and organs-at-risk (OARs). This comparison with the current state-of-the-art should precede the integration of 3D CZT SPECT/CT imaging into the clinical practice to perform Lu-177 QSPECT imaging and support personalized dosimetry.

Hence, the primary aim of this study was to compare the quantitative imaging characteristics and dose uncertainties of a 3D CZT SPECT/CT system with a conventional Anger-type SPECT/CT system for Lu-177-PSMA-617 dosimetry. To achieve this, we compared the Spectrum Dynamics Veriton 3D CZT SPECT/CT system with a Siemens Symbia T16 SPECT/CT where we initially established an optimal and clinically applicable protocol for Lu-177 QSPECT imaging with both systems. Once the optimal clinical imaging protocol was established, we determined the corresponding SPECT image characteristics and applied the uncertainty model for molecular radiotherapy [7], as proposed by the European Association of Nuclear Medicine (EANM) guideline, to evaluate Lu-177-PSMA-617 dose uncertainties based on both phantom measurements and clinical data.

## Materials and methods

### NEMA IQ phantom

The National Electrical Manufacturers Association (NEMA) Image Quality (IQ) phantom featuring six fillable spheres with inner diameters of 10 mm, 13 mm, 17 mm, 22 mm, 28 mm, and 37 mm was filled with Lu-177 activity concentrations of 6.41 MBq/mL for the spheres and 0.53 MBq/mL for the background. As such, a 12.1 contrast ratio was achieved between spheres and the background to replicate clinical lesions. With these concentrations, a High-count (HC) scan was first emulated, followed by nine other scans with the same sphere-to-background ratio but with decreasing activity concentrations due to physical decay. As a result, the last scan was considered a Low-count (LC) scan with an activity concentration of 0.8 MBq/mL for the spheres and 0.06 MBq/mL for the background.

### Conventional SPECT/CT imaging

Conventional SPECT/CT imaging was performed with a dual-head Siemens Symbia T16 SPECT/CT system, equipped with medium-energy general-purpose parallel-hole collimators. Acquisition parameters of the CT scan were set to 130 kV, 30 mAs, and $0.97 \times 0.97 \times 5\hbox { mm}^3$ voxel size while the SPECT scanning protocol followed the protocol described by Marin et al. [8] with the following parameters: a $128\times 128$ matrix, voxel size of 4.79 mm, 32 views with 30 s per view, and an energy window centered at the 208 keV photopeak (with a 20% width) combined with a lower scatter window (with a 10% width). The projection data were decay and scatter corrected and reconstructed using Ordered Subsets Expectation Maximization (OSEM) [9]. To optimize the reconstruction protocol such that sphere-to-background ratios were as close as possible to the true ratio while keeping the noise level as low as possible, the number of iterations and subsets were gradually increased (4i4s, 8i8s, 16i16s and 24i16s) with 16 the highest possible number of subsets. In addition, default post-smoothing was performed with a Gaussian filter of 1 mm Full width half maximum (FWHM), with additional filtering being considered to further reduce the noise if necessary.

### 3D CZT SPECT/CT imaging

The ring-like CZT system is a 12-headed Veriton SPECT/CT system, manufactured by Spectrum Dynamics. This system comes with an integrated collimator made out of Tungsten and optimized for low-energy photons (70 to 200 keV). As it is not an externally applied collimator, it cannot be changed and all imaging needs to be done with this collimator. Since the energy range of this system is limited to 200 keV because of the limited stopping power of the 6 mm CZT crystal, the energy window was centered at the lower 112.9 keV photopeak (20% width) of Lu-177. Triple energy window (TEW) scatter correction was used with the lower scatter window centered at 90.3 keV (20% width) and the upper energy window at 135.5 keV (20% width). The main and scatter windows were fixed by the manufacturer, such that no further optimization was feasible. The StarGuide from GE Healthcare is a similar 3D CZT SPECT/CT and has a crystal of 7.25 mm. It can therefore utilise the 208 keV peak. A full analysis of this system for Lu-177 was performed by Danieli et al. [10].

The phantom was scanned with auto-contouring activated, as this is also how patient images are acquired. In addition, using auto-contouring increased the total number of counts by 10.6% compared to not using auto-contouring. To perform auto-contouring, aluminum foil was placed around the NEMA IQ phantom. This was done to prevent any collisions with the detector heads, as the auto-contouring system relies on capacitance proximity. Without the aluminum foil, the Veriton SPECT/CT was not able to detect the plastic edge of the phantom.

The clinical protocol was based on the protocol by Vergnaud et al. [11] where it was concluded that applying TEW scatter correction gave the highest recovery coefficients compared to including a detector response model in the reconstruction. As detector response modeling and scatter correction cannot be combined, the latter was chosen. For the attenuation correction, a CT scan was performed at 120 kV, 21 mAs and $1.27 \times 1.27 \times 2.5\hbox { mm}^3$ voxel size. A voxel size of 2.46 mm was chosen for all SPECT reconstructions while reconstructed images were by default post-smoothed with a Gaussian filter of 3 mm FWHM. To optimize the reconstruction in terms of sphere-to-background ratios and noise level, the number of iterations was gradually increased (5, 10, 25, 50 and 100) while 2 subsets were chosen for each number of iterations to keep the number of subsets as low as possible to ensure convergence.

Once the optimal reconstruction protocol was determined, the impact of further reducing the acquisition time on image quality was also assessed. For this purpose, additional phantom scans with a shorter scanning time were obtained from the phantom data with 6 min of acquisition time by reducing the list mode data based on a shorter virtual scanning time. These virtual scans with a reduced number of counts were combined with the scans acquired with lower activity concentrations and compared to the 6 min scan which was considered as the reference high-count (HC) scan.

### SPECT image characteristics using the NEMA IQ phantom

#### Image calibration

**Fig. 1:**
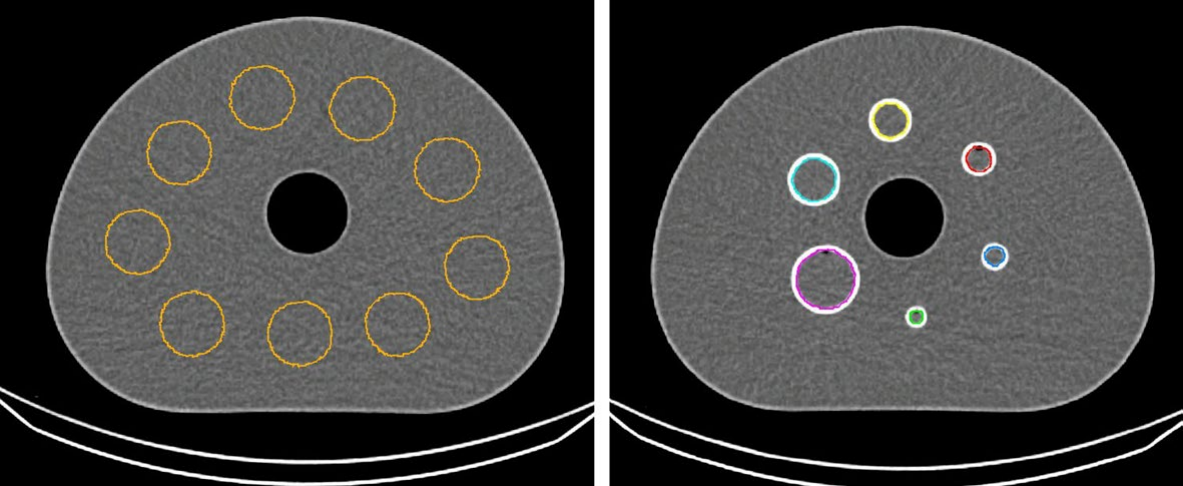
Delineation of the background VOI (left) and the spheres (right) on the corresponding CT image

To calibrate the SPECT system, nine spheres (each with 35 mm diameter) were drawn at different locations in the uniform region of the NEMA IQ phantom and used to compile one larger background Volume of Interest (VOI) (see Fig. 1) to measure the total number of counts $C_{total}$ in this background region.

For the conventional system, the calibration factor $Q_{conv}$, expressed in [cps/MBq], was calculated by Eq. 1 with $C_{total}$ the total number of counts in the predefined background, $A_{cal}$ the true decay-corrected activity in that specific VOI as measured with the radionuclide calibrator and *T* the total acquisition time.

1 $$\begin{aligned} Q_{conv} = \frac{C_{total}}{A_{cal}\cdot T} \end{aligned}$$

For a setup of 32 views per head and 30 s acquisition time per view, the total acquisition time equals $T = 32\cdot 30\,\text {s} \cdot 2$ since two detector heads were used. Because $C_{total}$ is defined as the total number of counts measured in all voxels of the VOI, the calibration factor $Q_{conv}$ depends on the acquisition time, reconstruction settings (voxel size and number of iterations and subsets) and amount of post-smoothing since all these factors can impact the total number of reconstructed counts. Therefore, a calibration factor should be determined for each predefined acquisition and reconstruction protocol.

For the 3D CZT system, the base unit for measurements is $\frac{PROPCPS}{mm^3}$, which is a proportional unit that considers the isotope’s physical characteristics for the detector sensitivity correction map and the time each voxel in the image was measured according to the system model. The latter is a result of the swiveling motion of the detectors, which makes acquisition time spatially dependent. The system calibration factor $Q_{czt}$ converts this proportional unit to Bq/mL which has the advantage that the calibration factor is independent of the acquisition time and voxel size. However, the system calibration is still dependent on the reconstruction parameters as these directly influence the activity concentration measured by the system. As a result, the calibration factor $Q_{czt}$ of the 3D CZT system is given by Eq. 2, with $C_{total}$ the total number of counts in the background VOI which is proportional to Bq/mL, $A_{cal}$ the true activity concentration in the background VOI expressed in MBq/mL and $10^6$ a correction factor to account for the difference in units.

2 $$\begin{aligned} Q_{czt} = \frac{C_{total}}{A_{cal}\cdot 10^6} \end{aligned}$$

Based on Eqs. 1 and 2, the relative uncertainty of the calibration factor *Q* is a combination of the uncertainty of the relative uncertainty on the radionuclide calibrator measurements $A_{cal}$ and the background count measurements $C_{total}$. This is given in Eq. 3. This dependence is visualized in the total uncertainty scheme in Fig. 3.

The relative uncertainty on the background count measurement was defined as the standard deviation divided by the average value of $C_{total}$, calculated over all ten scans (see Sect. ). The relative uncertainty on the radionuclide calibrator measurements was considered equal to the uncertainty of the activity measurement of the calibration vial.

3 $$\begin{aligned} \left[ \frac{u(Q)}{Q}\right] ^2 = \left[ \frac{u(C_{total})}{\bar{C}_{total}}\right] ^2 + \left[ \frac{u(A_{cal})}{A_{cal}}\right] ^2 \end{aligned}$$

#### Image quality assessment

To assess image quality, only the HC and LC scans were used. Each fillable sphere within the NEMA IQ phantom was defined on the corresponding CT image using a region-growing algorithm, with the edge of each sphere serving as a boundary (see Fig. 1). These VOIs were then transferred to the SPECT data. Due to the limited spatial resolution of the SPECT data, the two smallest spheres were excluded from the analysis.

For the 3D CZT and conventional SPECT/CT system, various reconstruction protocols were then considered. These protocols were evaluated based on the measured sphere-to-background ratios using the mean values of the spheres relative to the mean value of the composite background VOI, as a function of the background Coefficient of Variation (CoV). The goal was to identify the reconstruction settings that matched the true sphere-to-background ratio of 12.1 as closely as possible while keeping the background CoV (noise) as low as possible.

#### Image resolution

A matching filter method was used to estimate the spatial resolution of the high-count (HC) SPECT images that were acquired and reconstructed according to the optimized clinical SPECT imaging protocol [12].

For this approach, a template of the NEMA IEC phantom was generated starting from the HC SPECT/CT scan. First, the different spheres, the background and the lung compartment were delineated based on the corresponding CT data using region growing. For the Siemens Symbia PET/CT data, both the template and the HC SPECT scan were resampled to match the voxel size of the Veriton (2.46 mm), as the original voxel size of 4.79 mm resulted in undersampling of the VOIs from the CT image when transferred to the SPECT image. Next, the value of the background compartment of the template was set to the mean value of the background VOI of the HC SPECT image while the value of the spheres was set to 12.1 times the background value and the value of the lung compartment was set to zero. This was done to allow the template to match the SPECT scan as much as possible. Subsequently, the template was smoothed with a 3D isotropic Gaussian filter using different FWHM values, ranging from 0 to 18 mm.

For every FWHM, the Peak Signal-to-Noise Ratio (PSNR) was calculated using the HC SPECT scan as a reference. First, the Mean Squared Error (MSE) was computed between the reference HC SPECT image and the smoothed template image for all image slices containing the spheres. Next, the PSNR was calculated as the ratio between the maximum uptake value of the reference HC SPECT image and the MSE. This ratio is then converted to dB by Eq. 4.

4 $$\begin{aligned} PSNR = 20\,\log _{10}\left( \frac{MAX}{\sqrt{MSE}}\right) \end{aligned}$$

**Fig. 2:**
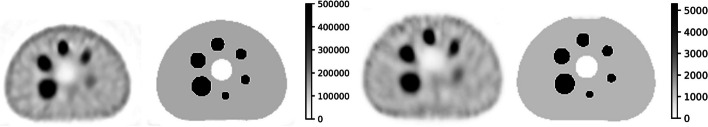
Axial view of the Veriton high-count SPECT scan of the NEMA IQ phantom, reconstructed with the optimized clinical protocol and its corresponding template for the same color window (left). Axial view of the Symbia high-count SPECT scan and its corresponding template for the same color window (right)

If smoothing of the template with a specific FWHM provided a voxelwise better approximation of the reconstructed HC SPECT image, the MSE would be lower for this FWHM and the corresponding PSNR higher compared to template images with a different degree of smoothing. To determine the FWHM resulting in the highest PSNR, the PSNR curve was approximated by a 2nd-order function in the relevant range and the FWHM corresponding to the highest PSNR value was determined by finding the root of the function. This FWHM was then considered the most representative value for the spatial resolution of the reconstructed SPECT image (Fig. 2).

### Dose uncertainty analysis

For clarification, a brief overview is given of the dosimetry uncertainty model used for this study. For more details, we refer to the EANM guideline on the uncertainty analysis for molecular radiotherapy absorbed dose calculations [7]. For $\beta$-emitters such as Lu-177, the Medical Internal Radiation Dose (MIRD) formalism defines the self-absorbed dose $\overline{D}$ in OAR and tumoral lesions as the product of the time-integrated activity $\tilde{A}$ and the corresponding tumor or OAR *S*-factor. The relative uncertainty of the absorbed dose $u(\overline{D}) / \overline{D}$ can therefore be calculated by Eq. 5, where the last term is the covariance term.

5 $$\begin{aligned} \left[ \frac{u(\overline{D})}{\overline{D}} \right] ^2=\left[ \frac{u(\tilde{A})}{\tilde{A}} \right] ^2 + \left[ \frac{u({S})}{{S}} \right] ^2 + 2 \frac{u(\tilde{A},S)}{(\tilde{A},S)} \end{aligned}$$

This equation can be expanded to Eq. 6. Here, *Q* represents the calibration factor and is calculated as mentioned in Sect. .

6 $$\begin{aligned} \begin{aligned} \left[ \frac{u(\overline{D})}{\overline{D}} \right] ^2&= \left[ \frac{u(Q)}{Q} \right] ^2 + \left[ \frac{u(R)}{{R}} \right] ^2 + \left[ \frac{u(C_i)}{C_i} \right] ^2 - \frac{\varphi }{R^2 V} \frac{\delta R}{\delta v} u^2(V) + |c_2|^2 \left[ \frac{u(V)}{V} \right] ^2 \\&\quad -2\frac{c_2}{RV} \left( \frac{\varphi }{2V}-\frac{\delta R}{\delta V} \right) u^2(V) \end{aligned} \end{aligned}$$

The volume uncertainty *u*(*V*) is calculated as in Eq. 7. Here, d is the equivalent diameter of the VOI and the uncertainty of this diameter is a combination of the voxel size *a* and the resolution quantified by the corresponding *FWHM*, as shown in Eq. 8. The S-factor uncertainty *u*(*S*) is directly proportional to *u*(*V*) as given in Eq. 9 with $c_2$ a fitting constant of the S-factor.

7 $$\begin{aligned} \left[ \frac{u(V)}{V} \right] ^2= & {} \left[ 3\frac{u(d)}{d} \right] ^2 \end{aligned}$$

8 $$\begin{aligned} {u^2(d)}= & {} \frac{a^2}{6} + \frac{\text {FWHM}^2}{4 ln(2)} \end{aligned}$$

9 $$\begin{aligned} \left[ \frac{u(S)}{S} \right] ^2= & {} \left[ c_2\frac{u(V)}{V}\right] ^2 \end{aligned}$$

The uncertainty of the recovery coefficient *u*(*R*) can be calculated by Eq. 10, assuming that *b* are adjustable parameters of the recovery coefficient function, $\textbf{g}_{\textbf{b}}$ is the vector containing the partial derivatives of the first order of *R* to *b* and $\textbf{V}_{\textbf{b,v}}$ is the covariance matrix that includes uncertainties of the fitting parameters **b** and *u*(*V*).

10 $$\begin{aligned} \left[ \frac{u(R)}{R} \right] ^2= \textbf{g}_{\textbf{b}}^T \textbf{V}_{\mathbf {(b,v)}} \textbf{g}_{\textbf{b}} \end{aligned}$$

The count rate uncertainty ${C_i}$ of the selected VOI at scan *i*, is calculated as in Eq. 11 with ${\varphi }$ the error function.

11 $$\begin{aligned} \left[ \frac{u(C)}{C} \right] ^2 = \left[ \frac{\varphi }{2R}\frac{u(V)}{V}\right] ^2 \end{aligned}$$

**Fig. 3:**
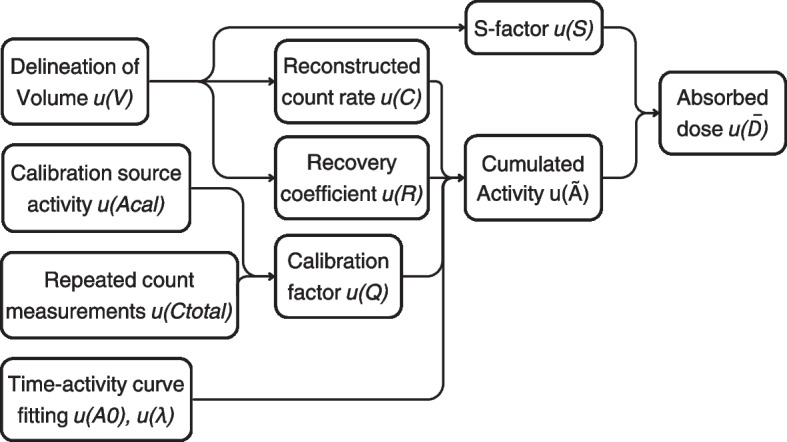
Flow diagram that shows the propagation of uncertainty

For clarification, the dependency of the absorbed dose on different factors is also visualized in Fig. 3.

It can be seen that the uncertainty associated with fitting the time-activity curves is omitted from the equations. Our current clinical practice involves Lu-177 QSPECT/CT imaging at two time points, 20 h and 168 h post-injection. Using a mono-exponential model for estimating the effective half-life, induces no uncertainty on the estimated effective half-life and initial activity.

The volumes of kidneys and different tumoral lesions were estimated based on patient data to translate the impact of the dose uncertainties to clinically relevant lesions and organ volumes. 27 QSPECT/CT scans were taken on both systems from 13 patients treated with Lu-177-PSMA-617. The left and right kidneys are delineated on all images and the mean volume and counts are used to make an average single kidney. For the tumoral estimates, five lesions were used that were visible on CT. All kidneys and tumoral lesions were delineated on the CT data using a region-growing algorithm to estimate the organ and lesion volume and to apply the appropriate recovery coefficient. However, for a lung metastas is that was also included, the CT-based delineation was adjusted based on the SPECT data to account for the additional smoothing because of breathing motion.

Next, the time-integrated activity coefficients (or residence time) of Lu-177-PSMA-617 in each of the lesions and kidneys were estimated by fitting a mono-exponential model through the two time points and dividing the area under the curve with the total injected activity. By fitting S-factor data against mass, a suitable S-factor was determined [13].

## Results

### SPECT Image quality assessment

**Fig. 4:**
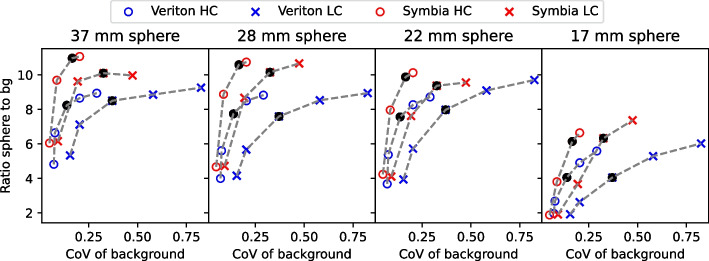
Sphere-to-background ratios for the NEMA IQ phantom for the 3D CZT Veriton SPECT/CT images in blue, and for the conventional Symbia SPECT/CT images in red (with the true sphere-to-background ratio equal to 12.1) The high-count (HC) images are depicted by ’o’ and the low-count (LC) images by ’x’. The increasing number of updates are 10, 20, 50, 100 and 200 for the 3D CZT Veriton SPECT reconstructions and 16, 64, 256 and 384 for the Symbia reconstructions. Data points were subsequently connected by the grey dotted line with black circles indicating the chosen clinical protocol for both systems

Figure 4 shows the sphere-to-background ratio (using the mean count values) versus background CoV for the largest four spheres for both systems.

For Veriton SPECT reconstruction with 50 updates, the mean difference from the true 12.1 sphere-to-background ratio, averaged over the four largest spheres for both the HC and LC scan, was 41.9%, while for Symbia SPECT reconstructions with 256 updates, this was 23.4%. No additional filtering was applied to the reconstructed images compared to the proposed reconstruction schemes for both systems in Sect. .

Furthermore, the difference between the ratios of the HC and LC scan was 2.7% for Veriton reconstructions and 5.0% for Symbia reconstructions. Meanwhile, the background CoV for the HC and LC SPECT data was 0.14 and 0.37 respectively for the Veriton SPECT reconstructions, and 0.17 and 0.33 respectively for the conventional Symbia SPECT reconstructions. This corresponded to an increase of the background CoV by a factor of 2.7 when considering LC instead of HC Veriton SPECT data, while the background CoV increased only by a factor of 1.9 when considering LC instead of HC Symbia SPECT data. As a result, Symbia SPECT images approximated the true activity ratio better by 18.5% compared to the Veriton SPECT images for the same level of background CoV while the noise difference between HC and LC SPECT data was also smaller for the Symbia system compared to the Veriton system.

Increasing the number of updates to 100 for the Veriton reconstructions, results in a 7.6% higher accuracy with a 35.3% mean difference from the true sphere-to-background ratio compared to a 42.9% mean difference for 50 updates. However, a reconstruction with 100 updates also resulted in a 5.1% difference in sphere-to-background ratio between the HC and LC SPECT data which was almost two times higher than for 50 updates. In addition, the noise, estimated as the background CoV, of HC and LC images reconstructed with 100 updates was 0.29 and 0.82 respectively which was around two times higher than for the images reconstructed with 50 updates. Therefore, Veriton reconstructions with 50 updates were considered as the optimal balance between resolution recovery and noise.

For the Symbia reconstructions, increasing the number of updates to 384 resulted in a mean difference to the true sphere-to-background ratio of 20.7% instead of 23.4% for images reconstructed with 256 updates. Reconstructions with 384 updates also resulted in a 6.8% difference in the true sphere-to-background ratio between HC and LC SPECT data and a background CoV of 0.2 and 0.47 for the LC and HC SPECT images respectively which were slightly worse values compared to reconstructions with 256 updates. Therefore, as a further increase in the number of updates did not significantly improve the sphere-to-background ratios while it resulted in additional noise, 256 updates were considered as the optimal choice for Symbia SPECT reconstructions.

To optimize the number of subsets for a given number of updates, we wanted to avoid using too many subsets because differences between reconstructions of subsets will increase with an increasing number of subsets such that a given reconstruction will be further away from the maximum-likelihood solution [14]. For the Veriton system, reconstructions using 6 iterations and 8 subsets (48 updates in total) resulted in a mean difference in the sphere-to-background ratio of only 1.99%, compared to a reconstruction using 25 iterations and 2 subsets, averaged over both the HC and LC scan. For the Symbia system, a high number of updates was needed to reach convergence. To allow reconstructions within a clinically acceptable time frame, a number of 16 subsets was chosen. This was the highest number of subsets available within the Siemens SPECT reconstruction software.

As a result, all further image analysis in this study, if not mentioned otherwise, was performed with SPECT reconstructions using 6 iterations and 8 subsets (6i8s) and 3 mm FWHM Gaussian post-smoothing for the Veriton system, and SPECT reconstructions using 16 iterations and 16 subsets (16i16s) and 1 mm FWHM Gaussian post-smoothing for the Symbia system.

### SPECT image calibration

For the conventional Symbia system, a *Q* factor of 11.12 cps/MBq was determined for a voxel size of 4.79 mm. This corresponded to a *Q* factor of 1.23 $\text {(cps/voxel)}/\text {(MBq/mL)}$ which is independent of the voxel size. The relative uncertainty was 4.35% for $C_{total}$ and 6.63% for *Q* with the inclusion of the uncertainty of $A_{cal}$ which was estimated as equal to 5% based on the uncertainty of the ordered calibration vial. For the chosen 3D CZT SPECT protocol, the unitless *Q* factor was 0.232 with a relative uncertainty of 2.99% and 5.83% for $C_{total}$ and *Q* respectively.

**Fig. 5:**
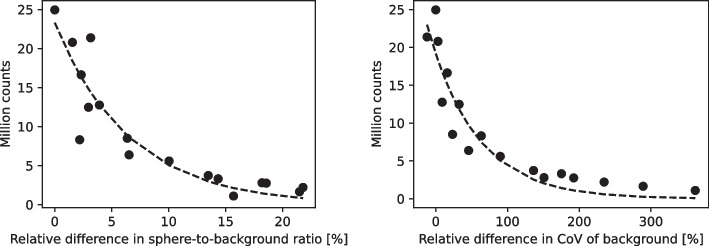
The effect of shortening the acquisition time for the Veriton system. SPECT data of a NEMA IQ phantom scan with 24.9 million counts was used as a reference. (Left) The relative absolute difference of the mean sphere-to-background ratios compared to the reference scan and calculated over the four largest spheres, increased exponentially with a decreasing number of counts or reduced scanning time. (Right) Reducing the scanning time and thus reducing the number of counts resulted in an exponential increase of the background CoV, relative to the reference scan

Reducing the scan time resulted in an increased absolute difference from the mean sphere-to-background ratio, averaged over the four largest spheres, of a reference scan with 24.9 million counts acquired during the full 8 min scanning time (as presented in Fig. 5). For this reference scan, the mean sphere-to-background ratio averaged over the four largest spheres, was 6.1. The curve mapping this relative absolute difference from the reference mean sphere-to-background ratio to the number of counts followed an exponential model where each doubling of the number of counts, corresponded to a 4.5% decrease in difference from the reference mean sphere-to-background ratio.

Additionally, using the same scan as a reference, the background CoV increased with a reduced number of counts (see Fig. 5). The curve mapping the relative difference from the reference background CoV to the number of counts was also fitted by an exponential model demonstrating that each doubling of the number of counts resulted in a decrease of relative difference from the reference background CoV by 47%.

Meanwhile, the calibration factor itself remained stable when reducing the acquisition time. Virtually reducing the acquisition time of the LC scan to two minutes resulted in a scan of 1.1 million counts. For this scan, the *Q* factor presented a difference of only 3.15% relative to the *Q* factor of the reference scan, whereas the background CoV was 4.6 times higher compared to the reference scan.

### SPECT image resolution

**Fig. 6:**
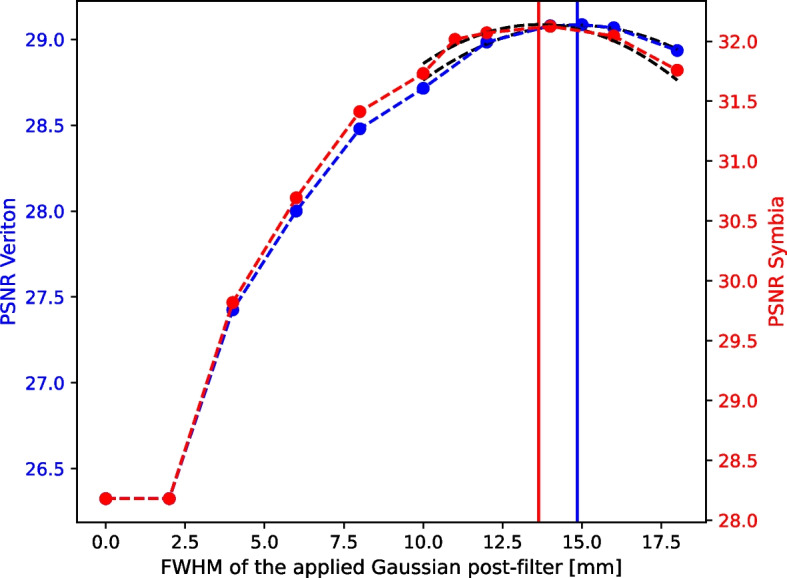
PSNR for the SPECT image and corresponding smoothed template as a function of the FWHM of the Gaussian filter used to smooth the template. The maximum PSNR identifies the most optimal FWHM value for the template to match the SPECT image resolution. On the left axis, the PSNR for a 6 min 3D CZT Veriton SPECT scan reconstructed with 6 iterations, 12 subsets and 3 mm FWHM Gaussian post-smoothing and on the right axis, the PSNR for the conventional 16 min Siemens Symbia SPECT scan reconstructed with 16 iterations, 16 subsets and 1 mm FWHM Gaussian post-smoothing

The results of the matching-filter method can be seen in Fig. 6 for the reconstruction protocols mentioned in Sect. . In addition, example SPECT images from both systems, together with the corresponding templates are presented in Fig. 2.

The spatial resolution, expressed as FWHM, was estimated at 14.8 mm for the Veriton system and at 13.6 mm for the Symbia system. It can also be seen that applying a filter on the template where the FWHM is smaller than the voxel size, has no effect. To verify that the templates were a good representation of the phantom scans, the recovery coefficients of the smoothed templates were compared to the ones of the corresponding SPECT scans. For the four largest spheres, an average relative difference of 19.3% was observed for the Veriton system and 13.6% for the Symbia system. The recovery coefficients are therefore in good correspondence and the template is a good approximation to evaluate the spatial resolution used in the dose uncertainty model.

### Dose uncertainty analysis

**Fig. 7:**
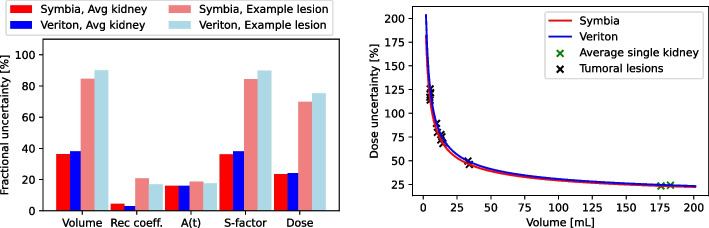
Fractional uncertainties of the different dosimetric parameters for an example lesion of around 14 mL and the average single kidney of around 176 mL for both the 3D CZT Veriton and Siemens Symbia system (left). The dependency of the dose uncertainty on the volume is visualized for both the 3D CZT Veriton and Siemens Symbia (right)

An overview of the numeric results of the uncertainty calculation for a middle-sized lesion of around 14 mL as an example can be found in Table 1. These values are also visualized in the left figure of Fig. 7, combined with the values of an average single kidney with a volume of around 176 mL. It can be seen that the volume uncertainty is slightly higher for the 3D CZT system with 86% uncertainty compared to 83% uncertainty for the conventional system.

For representative clinical lesion sizes, ranging from around 5 to 34 mL, that were identified based on the patient data, the volume uncertainty dominated the absorbed dose uncertainty. As shown in the dosimetry uncertainty scheme in Fig. 3, this was directly translated into the uncertainty for the S-factor and recovery coefficient. For the latter, it was thus important to calculate the uncertainty with the volume uncertainty included. This resulted in a 2.4 times higher relative uncertainty for the recovery coefficient for both systems and was used for calculating the uncertainty for the absorbed dose. Without the volume uncertainty, the uncertainty for the recovery coefficient was lower for the 3D CZT system compared to the conventional system, as was the uncertainty for the calibration factor. Combining all uncertainties resulted in a slightly higher dose uncertainty for the 3D CZT system compared to the conventional system.

In the right figure of Fig. 7 the dependency of the dose uncertainty on organ or lesion volume is given. This was done for the average single kidney of 176 mL and all five patient lesions with volumes ranging from 5 to 34 mL. These data confirmed that volume is the dominant factor driving the uncertainty for smaller structures or lesions. For infinitely large volumes, the analytical curve was shown to be asymptotic to 6.6% uncertainty for the conventional system and 5.8% uncertainty for the 3D CZT system. For an average single kidney with a volume of 176 mL, the volume and dose uncertainty were 36.2% and 23.5% respectively for the conventional system and 38.1% and 24.2% for the 3D CZT system. Thus, even for the average single kidney, the volume is still the dominant factor driving the uncertainty.

**Table 1:** Overview of the uncertainty values for the middle-sized lesion

	Symbia	Veriton
Voxel size (cm)	0.479	0.246
Spatial resolution in FWHM (mm)	14.80	13.60
Volume of lesion on CT (mL)	14.56	13.84
u(V)/V (%)	83.2	86.1
S-value	0.00556	0.00585
u(S)/S (%)	83.0	89.9
$u(C_{ref})/C_{ref}$ (%)	4.35	2.99
$u(A_{cal})/A_{cal}$ (%)	5.0	5.0
u(Q)/Q (%)	6.6	5.8
u( R)/R (%)	8.2	6.9
u(R )/R (u(V) included) (%)	20.1	16.9
u(A)/A (%)	18.9	17.6
Dose (Gy)	15.95	20.40
u(D)/D (%)	68.2	75.30
Dose range (Gy)	[5.08, 26.82]	[5.04, 35.76]

## Discussion

This study aimed to compare the dose assessment capabilities of a next-generation 3D CZT SPECT/CT (Veriton, Spectrum Dynamics) with those of a conventional SPECT/CT (Symbia T16, Siemens) using a dose uncertainly model to link image characteristics with clinically relevant dosimetric parameters. First, clinical imaging protocols were developed for both systems to allow quantitative SPECT imaging with similar noise characteristics for a fair comparison. For the Symbia, the reconstruction protocol was based on literature data presented by Marin et al. [8], which included detector response modeling and scatter correction. However, the reconstruction settings of Marin et al. were chosen on a combination of image parameters (including sphere-to-background ratios and noise levels) and visual assessment. This study focused on the dosimetric accuracy of the SPECT image data. As a result, the FWHM of the Gaussian post-filter was further optimized and changed from 12 mm to 1 mm to prioritize the sphere-to-background ratios.

For the Veriton system, reconstruction settings were based on the protocol proposed by Vergnaud et al. [11] who concluded that applying scatter correction was more important for maximizing recovery coefficients compared to including the detector response model in the reconstruction. Since detector response modeling and scatter correction cannot be combined in a single reconstruction protocol, the Veriton reconstructions for this study included scatter correction and no detector response modeling. Only the number of iterations was changed from 12 to 6, while keeping the subsets at 8 and the Gaussian post-filter at 3 mm FWHM.

Comparing the NEMA IQ SPECT data showed that the 3D CZT Veriton system behaved completely differently in terms of acquisition and reconstruction parameters compared to the conventional Symbia SPECT/CT system. The sphere-to-background ratios reached convergence after 50 updates for the 3D CZT system, whereas this was after 256 updates for the conventional system. In addition, Veriton SPECT data were more susceptible to noise when compared with the Symbia SPECT data. Furthermore, it was seen that a doubling of the counts resulted in an almost 50% reduction in the background CoV for the Veriton SPECT data. This suggests that one could consider using a minimal count threshold to determine the optimal acquisition time for the Veriton to minimize the noise, particularly for quantitative Lu-177 SPECT/CT at later time points following the administration of Lu-177 RLT.

In general, it was anticipated that the 3D CZT system would offer a superior trade-off between sensitivity and resolution compared to a conventional Anger-type SPECT/CT system. However, the spatial resolution of the reconstructed Lu-177 SPECT images is slightly better for the conventional system. This resulted in slightly higher absorbed dose uncertainties for the 3D CZT system which contradicts the predicted 50% lower dose uncertainty for this new type of SPECT system compared to a conventional system [15]. This difference can be fully attributed to the lower difference in spatial resolution between the two systems in the reconstructed Lu-177 SPECT/CT images. The difference between 14.8 mm and 13.6 mm FWHM spatial resolution obtained for Lu-177 SPECT images with the Veriton and Symbia system respectively, is much smaller than the datasheet values of 4.5 and 7.5 mm spatial resolution for Tc99m SPECT imaging in air at 10 cm off center in the central plane of the field of view, as reported for the Veriton and Symbia system respectively. This could be explained by the detector response model not being included in the SPECT reconstructions for the Veriton system, while for the Symbia system both scatter correction and detector response modeling were included. In addition, Lu-177 SPECT imaging with the Veriton was performed using the 113 keV photopeak which has a much larger scatter fraction than the 208 keV photopeak because of the higher scattering of lower energy photons and the downscattering of higher energy photons. As a result, it can be challenging to achieve even better image quality in terms of resolution and noise for the 113 keV Lu-177 SPECT images compared to the 208 keV Lu-177 SPECT images acquired with the Symbia.

The spatial resolution of the reconstructed images was determined by the matching filter method. PSNR was chosen as a metric as it is commonly used for quantitative image comparison while offering a global evaluation independent of the specific task at hand. However, differences between the SPECT imaging and the matching template could still be observed, as shown in Fig. 2, which could be explained by the spatially varying SPECT image resolution throughout the field of view. As a result, the estimated image resolution should be considered as the best possible estimate for the whole image and not as the underlying true value that is locally applicable for each lesion or organ.

Based on the estimated SPECT imaging resolution, the model by Gear et al. was used to compare the dose uncertainties for both SPECT/CT systems. As image resolution is the main determining factor for dose uncertainties obtained with this model, the reconstruction protocol with the highest spatial resolution will always result in the lowest dose uncertainties. However, in clinical routine not all lesions are visible on CT, resulting in observer-dependent lesion segmentations. Therefore, future work should include the impact of different delineation methods by physicians, in addition to including the variability of activity estimates induced by image noise. Especially including image noise in the dose uncertainty model will better reflect the optimal trade-off that needs to be found between increasing image resolution and increasing noise level, which becomes especially important for smaller lesions.

Spatial resolution aside, the Veriton system provided nevertheless superior sensitivity with 6 min of acquisition time providing consistent quantitative SPECT/CT images with similar dose uncertainty and noise properties as the Symbia system using 16 min of acquisition time. This means that, with an axial coverage of 32 cm, the Veriton can cover 160 cm in 30 min of acquisition time, which is the scan length typically used in clinical routine to cover the body from the top of the head until the femoral heads. For the Symbia with an axial coverage of 38.7 cm, this is 77.4 cm in 32 min of acquisition time. Nevertheless, dose uncertainties remain high for small lesions on both systems with a 50% dose uncertainty for a 28 mL lesion on the Symbia system and for a 33 mL lesion on the Veriton system.

In terms of limitations, this study only considered 2 imaging time points for the mono-exponential time activity curve fitting as is now done in clinical routine. However, Peters et al. reported an additional 12.9% uncertainty for two time-point dosimetry when comparing quantitative Lu-177 SPECT imaging at 20 and 160 h after administration of Lu-177 PRLT to dosimetry using 5 imaging time points. An additional 21.2% uncertainty was reported for dosimetry using only single time-point quantitative Lu-177 SPECT imaging at 20 h [16]. While this study included only ten patients with metastatic hormone-sensitive prostate cancer receiving Lu-177 PRLT, findings were in good accordance with the study by [17] reporting around 20% uncertainty for twenty patients. As a result, this study still underestimated the uncertainties for absorbed dose estimates. However, as this underestimation is not linked to system parameters, the current comparison of a 3D CZT SPECT system with a conventional SPECT system in terms of dose uncertainties remains valid.

## Conclusion

An optimized imaging protocol for the 3D CZT Veriton SPECT/CT system with 6 min of acquisition time per bed position resulted in slightly higher dose uncertainties compared to a conventional Siemens Symbia SPECT/CT system using a clinical protocol with 16 min of acquisition time per bed position. As a result, the 3D CZT SPECT/CT system requires significantly shorter acquisition times for similar dose uncertainties as a conventional system which makes quantitative Lu-177 SPECT/CT imaging much more accessible for treatment concurrent dosimetry. Nevertheless, SPECT-based dose uncertainties remain high, especially for smaller lesions.

## Data Availability

The Python scripts and SPECT-CT datasets are available from the corresponding author on reasonable request.

## Abbreviations

CoV: Coefficient of variation
EANM: European association of nuclear medicine
FWHM: Full width half maximum
HC: High-count
IQ: Image Quality
LC: Low-count
mCRPC: Metastatic castration-resistant prostate cancer
MIRD: Medical internal radiation dose
MSE: Mean squared error
NEMA: National electrical manufacturers association
OAR: Organs-at-risk
OSEM: Ordered Subsets expectation maximization
PCa: Prostate cancer
PSMA: Prostate-specific membrane antigen
PSNR: Peak signal-to-noise ratio
QSPECT: Quantitative single photon emission computed tomography
RLT: Radio-ligand therapy
VOI: Volume of Interest
